# Commentary: Development and Validation of a Self-reported Questionnaire for Measuring Internet Search Dependence

**DOI:** 10.3389/fpubh.2017.00095

**Published:** 2017-04-25

**Authors:** Mark D. Griffiths

**Affiliations:** ^1^International Gaming Research Unit, Psychology Division, Nottingham Trent University, Nottingham, UK

**Keywords:** online addiction, Internet addiction, Internet search dependence, online search dependence, questionnaire of Internet search dependence

Despite being a controversial topic, research into a wide variety of online addictions has grown substantially over the last decade (1, 2). My own research into online addictions has been wide ranging and has included online social networking [e.g., Ref. (3)], online sex addiction (4), online gaming addiction [e.g., Ref. (5)], online shopping addiction (6), and online gambling addiction [e.g., Ref. (7)]. As early as the late 1990s/early 2000s, I constantly argued that when it came to online addictions, most of those displaying problematic behavior had addictions on the Internet rather than addictions to it (i.e., they were not addicted to the medium of the Internet but addicted to applications and activities that could be engaged in *via* the Internet) (8–11).

A recent paper by Wang et al. (12) described the development of the Questionnaire of Internet Search Dependence (QISD), a tool developed to assess individuals who may be displaying a dependence on using online search engines (such as *Google* and *Baidu*). The notion of individuals being addicted to using search engines is not new and was one of five types of Internet addiction outlined by Young (13) in her typology (and what she termed “information overload” and referred to compulsive database searching). Although I criticized the typology on the grounds that most of the types of online addict were not actually Internet addicts but were individuals using the medium of the Internet to fuel other addictive behaviors (e.g., gambling, gaming, day trading, etc.), I did implicitly acknowledge that activities such as Internet database searching could theoretically exist, even if I did not think it was a type of Internet addiction. As far as I am aware, the new scale developed by Wang et al. (12) is the first to create and to psychometrically evaluate an instrument to assess “Internet search dependence.” As noted by the authors:

Subsequently, we compiled 16 items to represent psychological characteristics associated with Internet search dependence, based on the literature review and a follow-up interview with 50 randomly selected university students… We adopted the six criteria for behavioral addiction formulated by Griffiths (i.e., salience, mood modification, tolerance, withdrawal, conflict, and relapse) (10).

Given the authors claimed that they used an early version of my addiction components model [i.e., Griffiths (10)] rather than the most recent formulation [i.e., Ref. (14)] to help inform item construction, I was obviously interested to see the scale’s formulated items. More specifically, if an individual was genuinely addicted to searching online databases, I would have expected to see all of my six criteria applied as follows:

- *Salience*—this occurs when searching Internet databases becomes the single most important activity in the person’s life and dominates their thinking (preoccupations and cognitive distortions), feelings (cravings), and behavior (deterioration of socialized behavior). For instance, even if the person is not actually searching the Internet, they will be constantly thinking about the next time that they will be (i.e., a total preoccupation with Internet database searching).
- *Mood modification*—this refers to the subjective experiences that people report as a consequence of Internet database searching and can be seen as a coping strategy (i.e., they experience an arousing “buzz” or a “high” or paradoxically a tranquilizing feel of “escape” or “numbing” when searching Internet databases).
- *Tolerance*—this is the process whereby increasing amounts of time searching Internet databases are required to achieve the former mood modifying effects. This basically means that for someone engaged in Internet database searching, they gradually build up the amount of the time they spend searching Internet databases every day.
- *Withdrawal symptoms*—these are the unpleasant feeling states and/or physical effects (e.g., the shakes, moodiness, irritability, etc.), which occur when an individual is unable to search Internet databases because they are ill, the Internet is unavailable, or there is no Wi-Fi on holiday, etc.
- *Conflict*—this refers to the conflicts between the person and those around them (interpersonal conflict), conflicts with other activities (social life, hobbies, and interests), or from within the individual themselves (intra-psychic conflict and/or subjective feelings of loss of control) that are concerned with spending too much time searching Internet databases.
- *Relapse*—this is the tendency for repeated reversions to earlier patterns of excessive Internet database searching to recur and for even the most extreme patterns typical of the height of excessive Internet database searching to be quickly restored after periods of control.

Of the 12 QISD items constructed, very few appear to have anything to do with addiction and/or dependence, but this is most likely due to the fact that the authors also used data collected from 50 participants to inform their items and not just the criteria in the addiction components model. However, relying heavily on input from their participants has resulted in a number of key features in addiction/dependence not being assessed (i.e., no assessment of salience, mood modification, conflict, relapse, or tolerance). A couple of items may peripherally assess withdrawal symptoms (e.g., “I will be upset if I cannot find an answer to a complex question through Internet search”) but not in any way that is directly associated with addiction or dependence. This may be because the authors’ conceptualization of “dependence” is more akin to “over-reliance” rather than traditional definitions of dependence.

While the QISD may be psychometrically robust, it appears to have little face validity and does not appear to assess problematic engagement in Internet database searching (irrespective of how addiction or dependence is defined). Based on the addiction components model, my own 6-item scale would simply incorporate the six following questions, which would have much greater face validity than any item currently found in the QISD:

(1) Internet database searching is the most important thing in my life.
(2) Conflicts have arisen between me and my family and/or my partner about the amount of time I spend searching Internet databases.
(3) I engage in Internet database searching as a way of changing my mood.
(4) Over time, I have increased the amount of Internet database searching I do in a day.
(5) If I am unable to engage in Internet database searching, I feel moody and irritable.
(6) If I cut down the amount of Internet database searching I do, and then start again, I always end up searching Internet databases as often as I did before.

## Author Contributions

The author wrote the commentary and was responsible for all content.

## Conflict of Interest Statement

The author declares that the research was conducted in the absence of any commercial or financial relationships that could be construed as a potential conflict of interest.

## References

[1] Griffiths MD Kuss DJ Billieux J Pontes HM. The evolution of internet addiction: a global perspective. Addict Behav (2016) 53:193–5. 10.1016/j.addbeh.2015.11.001 26562678

[2] Kuss DJ Griffiths MD Karila L Billieux J. Internet addiction: a systematic review of epidemiological research for the last decade. Curr Pharm Des (2014) 20:4026–52. 10.2174/13816128113199990617 24001297

[3] Andreassen CS Pallesen S Griffiths MD The relationship between excessive online social networking, narcissism, and self-esteem: findings from a large national survey. Addict Behav (2017) 64:287–93. 10.1016/j.addbeh.2016.03.006 27072491

[4] Griffiths MD Internet sex addiction: a review of empirical research. Addict Res Theory (2012) 20:111–24. 10.3109/16066359.2011.588351

[5] Pontes H Griffiths MD Measuring DSM-5 Internet gaming disorder: development and validation of a short psychometric scale. Comput Human Behav (2015) 45:137–43. 10.1016/j.chb.2014.12.006

[6] Andreassen CS Griffiths MD Pallesen S Bilder RM Torsheim T Aboujaoude EN. The Bergen Shopping Addiction Scale: reliability and validity of a brief screening test. Front Psychol (2015) 6:1374. 10.3389/fpsyg.2015.01374 26441749 PMC4584995

[7] Canale N Griffiths MD Vieno A Siciliano V Molinaro S Impact of internet gambling on problem gambling among adolescents in Italy: findings from a large-scale nationally representative survey. Comput Human Behav (2016) 57:99–106. 10.1016/j.chb.2015.12.020

[8] Griffiths MD Internet addiction: does it really exist? In: Gackenbach J, editor. Psychology and the Internet: Intrapersonal, Interpersonal and Transpersonal Applications. New York: Academic Press (1998). p. 61–75.

[9] Griffiths MD Internet addiction: Internet fuels other addictions. Student BMJ (1999) 7:428–9.

[10] Griffiths MD Internet addiction: fact or fiction? Psychol Bull Br Psychol Soc (1999) 12:246–50.

[11] Griffiths MD Internet addiction – time to be taken seriously? Addict Res (2000) 8:413–8. 10.3109/16066350009005587

[12] Wang Y Wu L Zhou H Xu J Dong G Development and validation of a self-reported questionnaire for measuring internet search dependence. Front Public Health (2016) 4:274 10.3389/fpubh.2016.00274 28066753 PMC5167696

[13] Young KS Internet addiction: evaluation and treatment. Student BMJ (1999) 7:351–2.

[14] Griffiths MD A ‘components’ model of addiction within a biopsychosocial framework. J Subst Use (2005) 10:191–7. 10.1080/14659890500114359

